# Upregulated heme biosynthesis increases obstructive sleep apnea severity: a pathway-based Mendelian randomization study

**DOI:** 10.1038/s41598-022-05415-4

**Published:** 2022-01-27

**Authors:** Heming Wang, Nuzulul Kurniansyah, Brian E. Cade, Matthew O. Goodman, Han Chen, Daniel J. Gottlieb, Sina A. Gharib, Shaun M. Purcell, Xihong Lin, Richa Saxena, Xiaofeng Zhu, Peter Durda, Russel Tracy, Yongmei Liu, Kent D. Taylor, W. Craig Johnson, Stacey Gabriel, Joshua D. Smith, François Aguet, Kirstin Ardlie, Tom Blackwell, Alexander P. Reiner, Jerome I. Rotter, Stephen S. Rich, TOPMed Sleep Traits Working Group, Najib Ayas, Deepika Burkardt, Brian Cade, Han Chen, Danielle Clarkson-Townsend, Joyita Dutta, Lynette Ekunwe, Caitlin Floyd, Sina Gharib, Matthew Goodman, Daniel Gottlieb, Einat Granot-Hershkovitz, Lauren Hale, Patrick Hanly, Scott Heemann, Chao Hsiung, Tianyi Huang, Anne Justice, Brendan Keenan, Jacqueline Lane, Jingjing Liang, Xihong Lin, Jiayan Liu, Noah Lorincz-Comi, Ulysses Magalang, Diego R. Mazzotti, Hao Mei, Julie Mikulla, Amy Miller, Miremad Moafi-Madani, Debby Ngo, Jeff O’Connell, Heather Ochs-Balcom, Allan Pack, Sanjay Patel, Shaun Purcell, Susan Redline, Richa Saxena, Rachel Soemedi, Tamar Sofer, Jae Hoon Sul, Shamil Sunyaev, Cynthia Tchio, Heming Wang, Ava Wilson, Lluvia Xia, Man Zhang, Hufeng Zhou, Xiaofeng Zhu, Susan Redline, Tamar Sofer

**Affiliations:** 1 Division of Sleep and Circadian Disorders, Brigham and Women’s Hospital, Harvard Medical School, 221 Longwood Ave BLI 252, Boston, MA 02115 USA; 2 Broad Institute of MIT and Harvard, Cambridge, MA 02142 USA; 3 Department of Epidemiology, Human Genetics and Environmental Sciences, School of Public Health, Human Genetics Center, The University of Texas Health Science Center at Houston, Houston, TX 77030 USA; 4 School of Biomedical Informatics, Center for Precision Health, The University of Texas Health Science Center at Houston, Houston, TX 77030 USA; 5 VA Boston Healthcare System, Boston, MA USA; 6 Department of Medicine, Computational Medicine Core, Center for Lung Biology, UW Medicine Sleep Center, University of Washington, Seattle, WA USA; 7 Department of Psychiatry, Brigham and Women’s Hospital, Harvard Medical School, Boston, MA 02115 USA; 8 Department of Biostatistics, Harvard T. H. Chan School of Public Health, Boston, MA USA; 9 Department of Statistics, Harvard University, Cambridge, MA USA; 10 Massachusetts General Hospital, Center for Genomic Medicine, Harvard Medical School, Boston, MA USA; 11 Department of Anesthesia, Critical Care and Pain Medicine, Massachusetts General Hospital, Harvard Medical School, Boston, MA USA; 12 Department of Population and Quantitative Health Sciences, School of Medicine, Case Western Reserve University, Cleveland, OH 44106 USA; 13 Department of Pathology and Laboratory Medicine, The Robert Larner, M.D. College of Medicine at the University of Vermont, Burlington, VT 05446 USA; 14 Divisions of Cardiology and Neurology, Duke University Medical Center, Durham, NC 27710 USA; 15 Department of Pediatrics, The Institute for Translational Genomics and Population Sciences, The Lundquist Institute for Biomedical Innovation at Harbor-UCLA Medical Center, Torrance, CA 90502 USA; 16 Department of Biostatistics, University of Washington, Seattle, WA 98195 USA; 17 Northwest Genomic Center, University of Washington, Seattle, WA USA; 18 Department of Biostatistics, University of Michigan School of Public Health, Ann Arbor, MI USA; 19 Department of Epidemiology, University of Washington, Seattle, WA USA; 20 Division of Public Health Sciences, Fred Hutchinson Cancer Research Center, Seattle, WA USA; 21 Center for Public Health Genomics, University of Virginia, Charlottesville, VA 22908 USA; 22 Department of Medicine, The University of British Columbia, Vancouver, Canada; 23 National Heart, Lung, and Blood Institute, Bethesda, USA; 24 Department of Electrical and Computer Engineering, University of Massachusetts Lowell, Lowell, USA; 25 University of Mississippi Medical Center, Jackson, MS USA; 26 Westat, Atlanta, USA; 27 Population and Preventive Medicine, Program in Public Health, Stony Brook Medicine, Stony Brook, USA; 28 Department of Medicine, University of Calgary, Calgary Alberta, Canada; 29 Westat, Rockville, USA; 30 National Health Research Institute Taiwan, Miaoli City, Taiwan, ROC; 31 Center for Biomedical and Translational Informatics, Geisinger Medical Center, Danville, USA; 32 Division of Sleep Medicine, Department of Medicine, University of Pennsylvania, Philadelphia, USA; 33 Division of Pulmonary, Critical Care and Sleep Medicine, Ohio State University, Columbus, USA; 34 Department of Internal Medicine, University of Kansas Medical Center, Kansas City, USA; 35 Department of Epidemiology, Brown University, Providence, USA; 36 Division of Pulmonary, Critical Care and Sleep Medicine, Beth Israel Deaconess Medical Center, Boston, USA; 37 School of Medicine, University of Maryland, Baltimore, USA; 38 Department of Epidemiology and Environmental Health, University at Buffalo, Buffalo, USA; 39 Division of Pulmonary, Allergy and Critical Care Medicine, University of Pittsburgh, Pittsburgh, USA; 40 Department of Psychiatry and Biobehavioral Sciences, UCLA, Los Angeles, USA; 41 Department of Medicine, University of Alabama Birmingham, Birmingham, AL USA

**Keywords:** Gene expression, Genomics

## Abstract

Obstructive sleep apnea (OSA) is a common disorder associated with increased risk of cardiovascular disease and mortality. Iron and heme metabolism, implicated in ventilatory control and OSA comorbidities, was associated with OSA phenotypes in recent admixture mapping and gene enrichment analyses. However, its causal contribution was unclear. In this study, we performed pathway-level transcriptional Mendelian randomization (MR) analysis to investigate the causal relationships between iron and heme related pathways and OSA. In primary analysis, we examined the expression level of four iron/heme Reactome pathways as exposures and four OSA traits as outcomes using cross-tissue *cis-*eQTLs from the Genotype-Tissue Expression portal and published genome-wide summary statistics of OSA. We identify a significant putative causal association between up-regulated heme biosynthesis pathway with higher sleep time percentage of hypoxemia (*p* = 6.14 × 10^–3^). This association is supported by consistency of point estimates in one-sample MR in the Multi-Ethnic Study of Atherosclerosis using high coverage DNA and RNA sequencing data generated by the Trans-Omics for Precision Medicine project. Secondary analysis for 37 additional iron/heme Gene Ontology pathways did not reveal any significant causal associations. This study suggests a causal association between increased heme biosynthesis and OSA severity.

## Introduction

Obstructive sleep apnea (OSA) is a common disorder affecting more than 20% of the middle aged and older population^1^. OSA is associated with an increased risk of multiple cardiometabolic diseases including hypertension, diabetes, coronary artery disease and mortality^2^. Despite its high prevalence and co-morbidities, current management and treatment approaches are limited, reflecting an incomplete understanding of the underlying molecular bases of the disorder.

Multiple lines of recent research suggest a novel role of iron/heme metabolism and related genes in the pathophysiology of OSA. Iron and heme metabolism influence a wide variety of biological systems relevant to OSA, including redox balance, inflammatory response, oxygen transport and energy metabolism, and may be causally associated with OSA through pathways that influence obesity, ventilatory control, or comorbidities^3–6^. Heme degradation regulated by *heme oxygenase 1* and *2* (*HMOX1* and *HMOX2*) and their products (including carbon monoxide oxygen and biliverdin) have been implicated in carotid body sensing to hypoxia^7^. Our prior admixture mapping of OSA in a Hispanic/Latino-American cohort identified that the heme biosynthesis gene ferrochelatase (*FECH*) was associated with the apnea hypopnea index (AHI) and overnight hypoxemia traits^8^. Gene set enrichment analyses in recent transcriptome-wide studies also identified that the heme metabolic pathway was associated with OSA traits and was up-regulated in individuals with more severe disease or in response to withdrawal of continuous positive airway pressure (CPAP) treatment^9,10^. It is therefore not clear whether the iron or heme related genes and pathways are causally associated with OSA, or rather are biomarkers of disease activity or severity.

Mendelian randomization (MR) is an inference method utilizing germline mutations as a natural experiment to investigate the causal relationship between an exposure and an outcome. There are three basic assumptions that need to be satisfied to ensure the validation of genetic instrument variables (IVs): (1) the IVs are strongly associated with the exposure; (2) the IVs are independent of unmeasured confounders for the associations between the IVs and outcome; and (3) the IVs are associated with the outcome exclusively through the effect on exposure (i.e., no horizontal pleiotropy)^11^. Recent MR analyses have provided evidence of causal associations between higher BMI with increased OSA risk, snoring, and excessive daytime sleepiness^12–14^. However, no MR study has interrogated transcriptional causal associations for OSA, which have been applied to other disease outcomes and revealed new molecular mechanisms^15^. In this study, we implement a novel pathway-based MR approach to investigate the causal relationship between expression levels of iron/heme related pathways.

## Methods

### Candidate iron/heme related pathways

**Figure 1:**
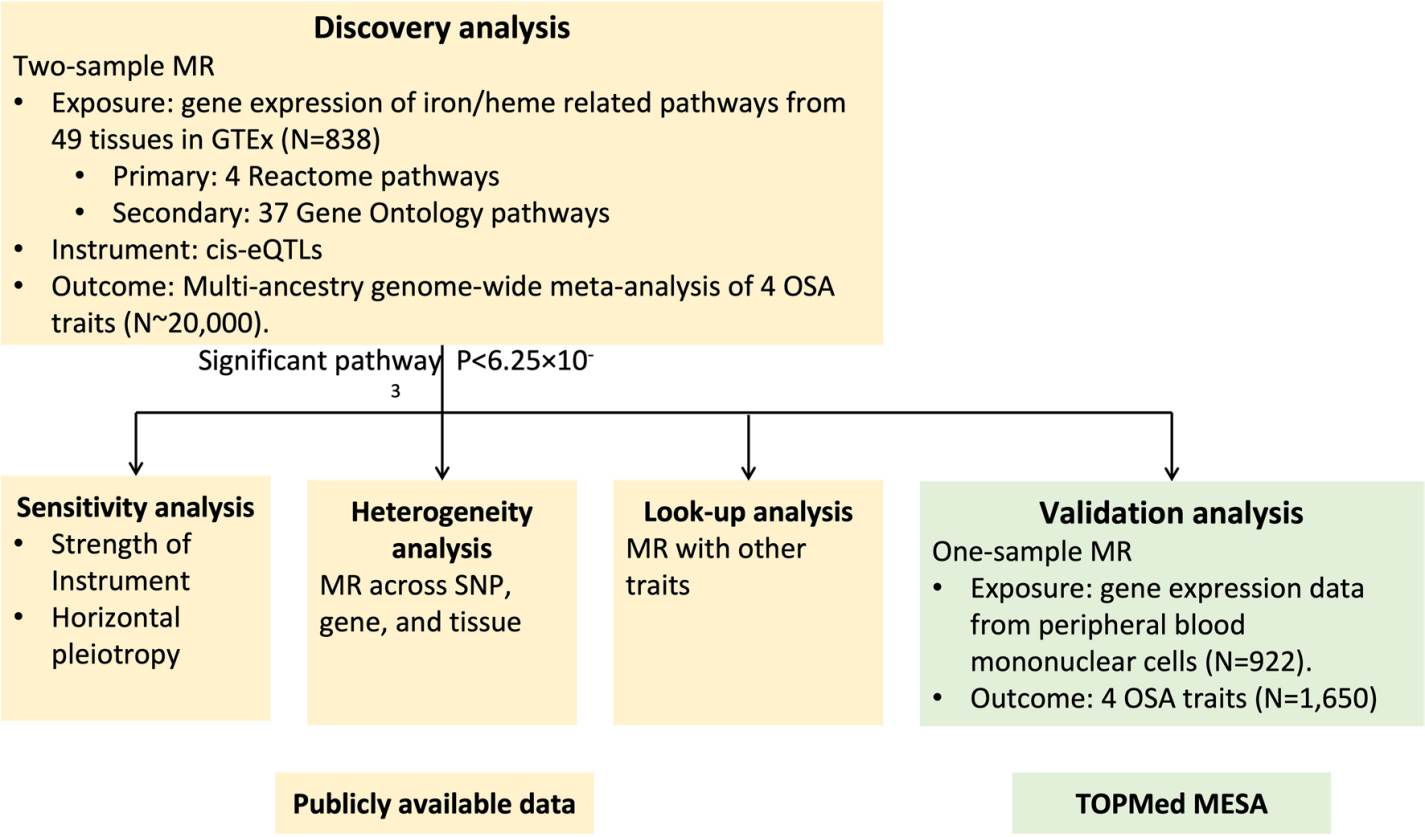
Analysis flow.

We used Molecular Signatures Database (MSigDB) v7.4^16,17^ to search for genes in iron/heme related pathways. Four canonical pathways derived from the Reactome database—heme biosynthesis, heme degradation, heme signaling, and iron uptake and transport pathways—were investigated in primary analysis. Thirty-seven Gene Ontology (GO) pathways containing the word “iron”, “heme”, or “hemoglobin” were also analyzed in secondary analysis. A complete list of candidate pathways and genes are summarized in Supplementary Table 1. Assuming that genes in a pathway act together and affect OSA uniformly, we investigated a pathway effect on OSA using the averaged expressions of genes as the exposure. The overall analysis flow is described in Fig. 1.

### Discovery analysis

The discovery analysis was performed using two-sample MR R package^18^ (https://mrcieu.github.io/TwoSampleMR/) and publicly available summary statistics from Genotype-Tissue Expression (GTEx) Portal^19^ (http://gtexportal.org/home/) and genome-wide association analyses (GWAS) of OSA traits^20,21^. The exposure and outcomes were measured in non-overlapping datasets, which minimizes false-positive discoveries^11,22^.

#### Exposure

We used a cross-tissue exposure effect, under the assumption that multiple physiological systems are involved OSA and can be estimated by averaging gene expression across multiple tissues. The exposure data were extracted from the gene expression data across 49 tissues in 838 donors from GTEx Portal (v8)^19^. The donors were comprised of 67.1% males and 84.6% whites, and 83.5% were aged 40–70 years old. A total of 347 iron/heme related genes were available. Local expression quantitative trait loci (cis-eQTLs; p < 10^–5^) within 1 Mb of the transcription start site for a gene in each available tissue were calculated adjusted for batch effect, sequencing platform, sequencing protocol (PCR-based or PCR-free), sex, and population structure, using publicly available data through GTEx.

#### Outcome

The outcome summary statistics were extracted from multi-ancestry GWAS meta-analysis of four key OSA traits including AHI and the metrics of sleep associated hypoxemia: average and minimal oxygen saturation (SpO_2_) during sleep and percent sleep time with SpO_2_ < 90%^20,21^. The total sample sizes varied from 19,733 to 22,736 and included 30–35% individuals of European ancestry background. Briefly, GWAS were performed on normalized OSA traits adjusting for sex, age, BMI (including interaction and quadratic terms), and population and family structure. Genome-wide summary statistics are available on the sleep knowledge portal (https://sleep.hugeamp.org).

#### Instrumental variables (IVs)

**Figure 2:**
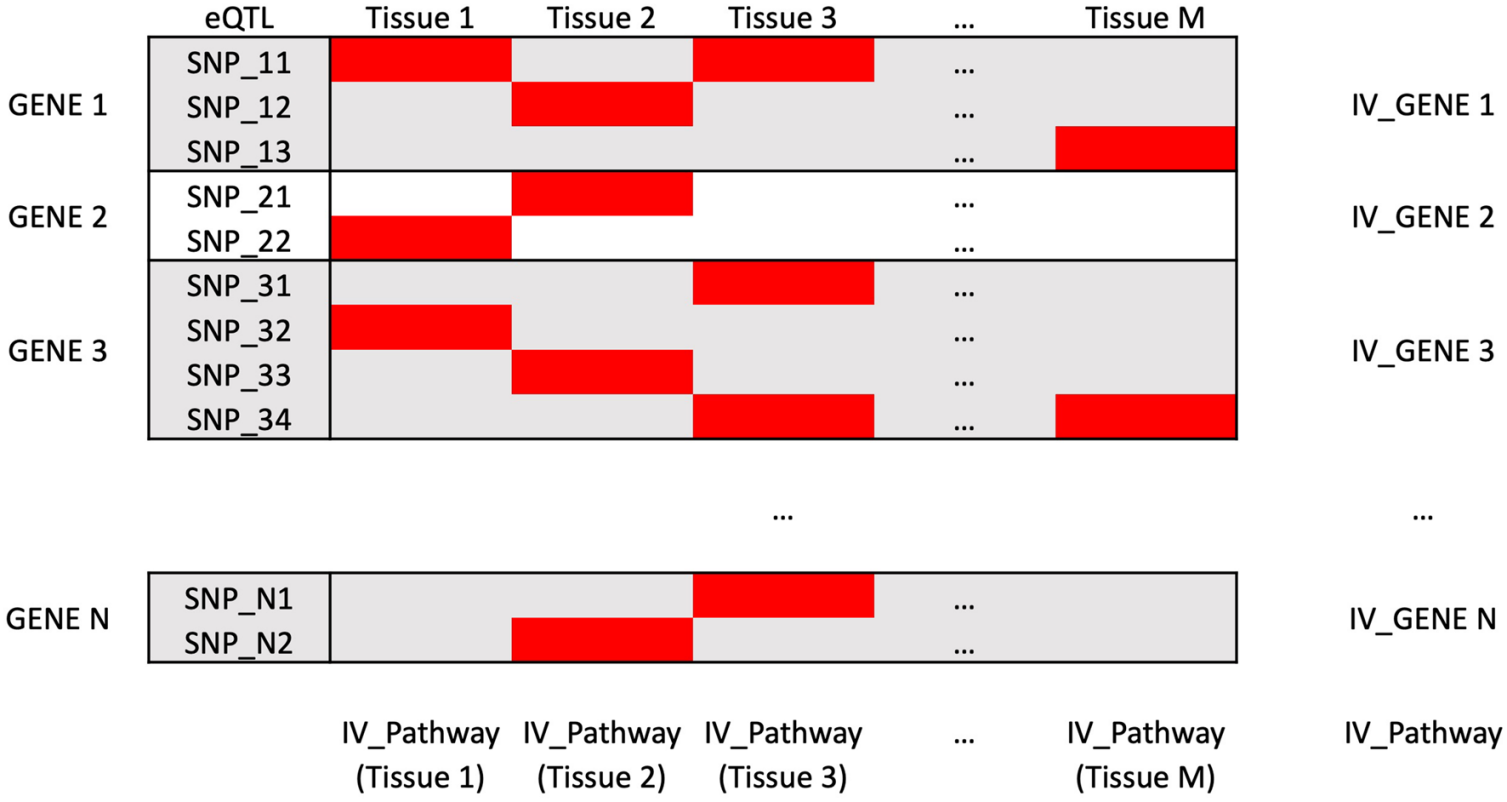
Illustration of the selection strategy of instrumental variables (IVs). The most significant cis-eQTLs in any tissues for a gene are highlighted in red.

The IVs for a given iron/heme related pathway were constructed as illustrated in Fig. 2. In brief cis-eQTL (with *p* value threshold < 10^–5^) in any gene from a pathway of interest (highlighted in red color in Fig. 2) were selected to mimic the tissue specific gene-level expressions. Of these, the most significant cis-eQTLs in any tissue for a gene were selected as IVs to mimic the cross-tissue overall gene-level expressions as a strategy for maximizing the instrument strength^23^. If a SNP was the most significant eQTL in more than one tissue (e.g., Fig. 2: SNP_11 in tissue 1 and 3), the cross-tissue SNP-gene association was calculated using the eQTL from the most significant tissue. We then selected the IVs of all available genes in a pathway to mimic an aggregate pathway-level expression effect, in which the exposure is the average cross-tissue expression across genes in the pathway (pathway effect defined earlier), i.e. under the assumption that $Y=(\sum_{j=1}^{p}{E}_{j}{\beta }_{j})+X\alpha + \varepsilon ={\beta }_{e}\times (\sum_{j=1}^{p}{\frac{{\beta }_{j}}{{\beta }_{e}}E}_{ij})+X\alpha + \varepsilon$, and ${E}_{ij}={\beta }_{s}SN{P}_{ij}+{x}_{i}{\alpha }_{j}+ {\varepsilon }_{ij}$, where ${E}_{j}$ is the expression level of the j-th gene in the pathway, ${\beta }_{e}$ is the average effect of gene expression in the pathway and ${\beta }_{j}/{\beta }_{e}$ represents potential heterogeneity of the effects of genes in the pathway, *X* and $\alpha$ are the covariate term and effect. We also selected IVs within each tissue (each column in Fig. 2) to evaluate the heterogeneity effect across tissues. Before conducting MR, we performed linkage disequilibrium (LD) based clumping (r^2^ < 0.01) to retain independent variants in all grouping strategies (gene, tissue, and pathway level). If a SNP IV was unavailable in the OSA outcome GWAS, we replaced it by proxy SNPs in high LD (r^2^ > 0.8).

#### MR, sensitivity and heterogeneity analyses

We performed MR between each iron/heme related pathway expression as exposures and the four OSA trait as outcomes using the TwoSampleMR package^18^ to investigate the potential causal effect. The inverse variance weighted (IVW) method, which has better power but assumes no horizontal pleiotropy, was performed as the primary test. The significance level was calculated as *p* value < 6.25 × 10^–3^ (accounting for two independent OSA traits, estimated based on eigenvalues of their correlations^24^, and four canonical pathways). Significant pathways were then followed up for a set of sensitivity and heterogeneity analyses. Note that this significance level is conservative given the pathway expression levels contain correlated data. The strength of IVs was evaluated by mean *F* statistic in the IVW test. $\overline{F }$ ≤ 10 indicates a violation of the strong instrument assumption. We performed MR Egger regression to assess potential horizontal pleiotropy. A significant departure (*p* < 0.05) of the MR-Egger intercept indicates presence of horizontal pleiotropy. MR-Egger slope was also tested as a sensitivity analysis in considering pleiotropic effects. The strength of IVs was evaluated by ${I}_{GX}^{2}$ statistic in MR-Egger^25^. ${I}_{GX}^{2}$ ≤ 0.9 indicates a violation of the strong instrument assumption. We investigated the heterogeneity of MR across IVs, genes, and tissues. The heterogeneity of SNPs in IVW was evaluated using Cochran’s Q statistics and leave-one-out analyses^11^. The SNP heterogeneity of the MR-Egger fit was evaluated using Rücker’s Q′ statistic (an extended version of Cochran’s Q statistic)^26^. Significant Q and Q′ suggests significant heterogeneity effects across IVs on the outcome. MR-PRESSO test was performed to detect (pleiotropic) IV outliers^27^. We also evaluated the heterogeneity effects across genes and tissues by performing MR restricted to genes and tissues in significant pathways using IVW (for number of SNP IVs ≥ 2) or the Wald ratio test (for single SNP IV). We further looked up the effect of the significant pathways by performing MR with other health outcomes including blood disorders, blood cell traits, and key comorbidities of OSA using GWAS summary statistics from IEU GWAS database (https://gwas.mrcieu.ac.uk/).

### Validation analysis

The significant pathway identified in discovery analysis (*p* < 6.25 × 10^–3^) was followed for validation using summary level one sample MR in the Multi-Ethnic Study of Atherosclerosis (MESA), a study that participated in the multi-ancestry OSA GWAS used in discovery analysis and collected omics data independent of sleep exam^20,21^. The MESA is a longitudinal study of risks for subclinical cardiovascular disease in multiple ethnic groups (including Asian American, African American [AA], European American [EA], Hispanic/Latino American [HA])^28^. In total, 6814 participants aged from 45 to 84 were recruited from 6 communities: Baltimore MD, Chicago IL, Los Angeles CA, New York NY, Minneapolis/St. Paul MN, and Winston-Salem NC at baseline in 2000 and then followed longitudinally, including an ancillary sleep exam in proximity to Exam 5 (2010–2013).

#### Instrumental variables

Whole-genome sequencing (WGS) and other omics (including epigenomics, transcriptomics, proteomics, and metabolomics) data were collected in 4595 individuals as part of the National Heart, Lung and Blood Institute (NHLBI) Trans-Omics for Precision Medicine (TOPMed) program^29^, with both WGS and RNA-seq available for 966 MESA participants. In this analysis, we used the Freeze 8 release WGS data sequenced at the Broad Institute (mean depth > 30×) and called by the TOPMed Informatics Resource Center at University of Michigan. All sequences were remapped to 1000 Genomes build 38 human genome references and annotated using a standard pipeline^30^. Variants with minor allele count < 2, minimum depth < 10× or missing rate > 5% were removed. Sample level quality control (QC) including Mendelian and sex concordance, and concordance with prior genotyping array data were performed. The same IVs used in discovery analyses were extracted for validation analyses.

#### Exposure

At MESA Exam 5, mRNA data were collected from peripheral blood mononuclear cell (PBMC), monocytes or T-cells. In this analysis we used gene level expression data from PBMCs. Quality of RNA samples was assessed using RNA Integrity Number (RIN, Agilent Bioanalyzer) before shipment to sequencing centers. PBMC samples were sequenced at the Broad Institute (N = 498), and at the North West Genomics Center (NWGC; N = 468) using harmonized protocols. Library preparation was performed using the Illumina TruSeq™ Stranded mRNA Sample Preparation Kit. RNA was sequenced as 2 × 101 bp paired-end reads on the Illumina HiSeq 4000 according to the manufacturer’s protocols. Information about the RNA-seq pipeline used for TOPMed can be found in https://github.com/broadinstitute/gtex-pipeline/blob/master/TOPMed_RNAseq_pipeline.md under MESA RNA-seq pilot commit 725a2bc. Overall, there were 922 individuals with gene-level expression data in PBMC who had complete covariate data (described below), including 180 AAs, 437 EAs, and 305 HAs.

#### OSA outcomes

OSA measures were collected in MESA for 2060 individuals who did not report regular use of oral devices, nocturnal oxygen, or nightly positive airway pressure devices using in-home unattended15-channel polysomnography (Compumedics Somte System, Abbotsford, AU) as described^31^. Sleep records were scored at a central Sleep Reading Center using standard criteria. In this paper, AHI was defined as the sum of all apneas plus hypopneas, each with a minimum 3% desaturation. Average and minimum SpO_2_ during sleep and sleep time percentage below SpO_2_ < 90% were extracted from the finger pulse oximetry recording during polysomnography. A total of 492 AAs, 698 EAs, and 460 HAs had WGS data, and 98 AAs, 200 EAs, and 164 HAs had both WGS and gene expression data.

#### MR analysis

We performed summary level one sample MR between the most significant iron/heme related pathways and OSA traits using the IVW method. Because we used the summary level IV-exposure-outcome association statistics in MESA, to increase statistical power we did not restrict our analyses to individuals with both expression and OSA measures. The IV-exposure associations were calculated in 922 individuals with WGS and gene expression data. Gene expression data were log transformed (after replacing counts of 0 with half the minimum observed values in the sample) and adjusted for age, sex, BMI, batch effects (shipment, plate, and sequencing site), study site, 11 genotype PCs, and race/ethnicity in analyses that included all individuals together. Genetic association analysis further used the fully-adjusted two-stage procedure for rank-normalizing residuals^32^. The IV-outcome associations were calculated in 1650 individuals with WGS and OSA measures. OSA traits were rank-normalized and adjusted for age, sex, BMI, study sites, genotyping PC, and race/ethnicity, via the fully adjusted two-stage procedure^32^. We also performed ancestry specific analyses restricted to AAs, EAs, and HAs respectively (without adjusting for ancestry).

### Ethics declarations

This work was approved by the Institutional Review Board of Mass General Brigham and complies with all relevant ethical regulations. All study participants provided informed consent.

## Results

### Discovery MR

**Figure 3:**
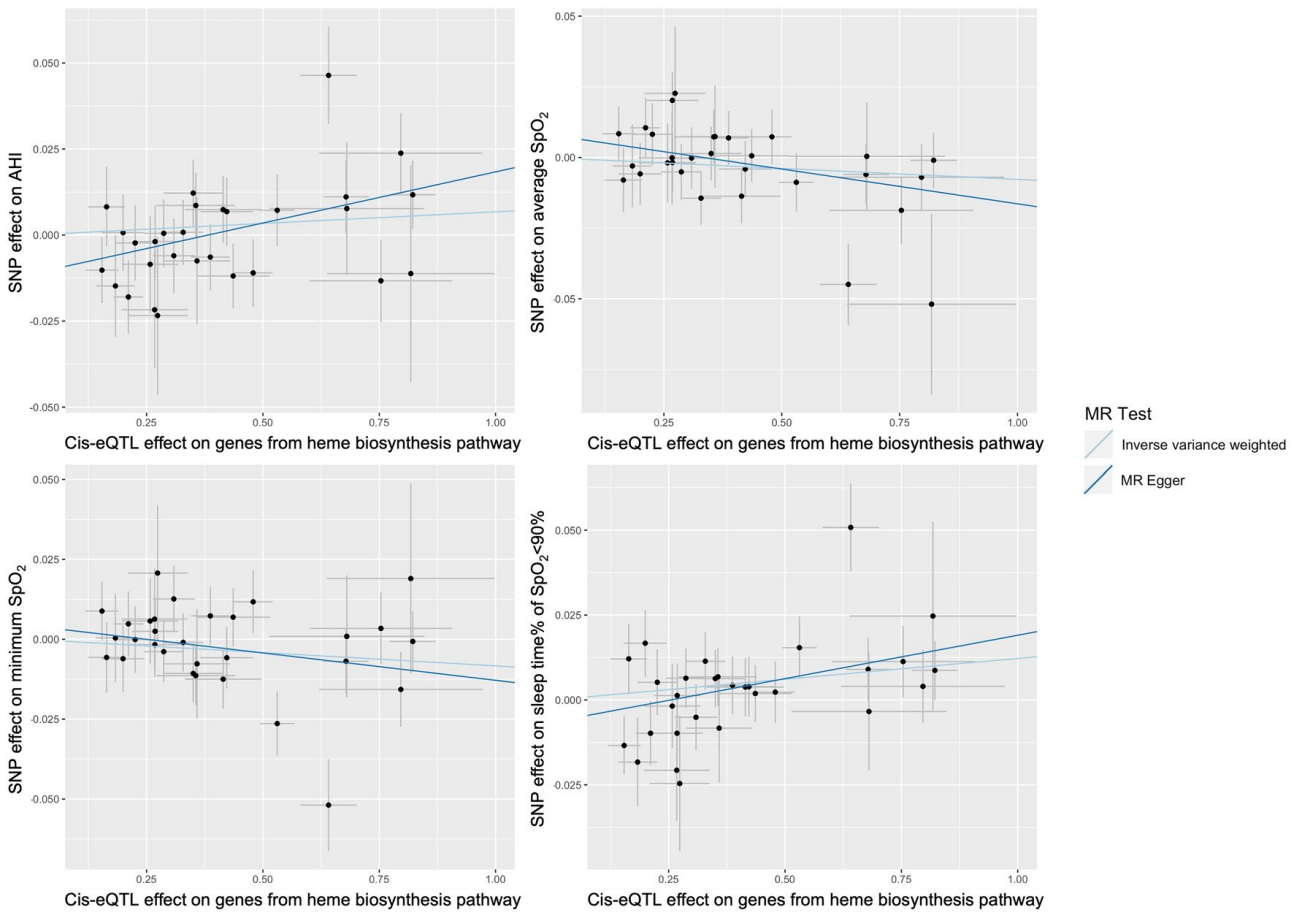
Scatter plots of SNP effects on the expressions of genes from Heme Biosynthetic Process pathway vs OSA traits in discovery analysis.

The results of the primary two-sample MR analyses between the exposures of each of the four canonical iron/heme related pathways and four OSA traits using IVW approach are summarized in Table 1. We identified a significant putative causal association between increased heme biosynthesis pathway expression and higher sleep time% of SpO_2_ < 90% ($\beta$ = 0.012, *p* = 6.14 × 10^–3^). Associations were also suggested between increased heme biosynthesis pathway expression with higher AHI ($\beta$ = 0.007, *p* = 0.189), lower average SpO_2_ ($\beta$ = − 0.008, *p* = 0.078) and minimum SpO_2_ ($\beta$ = − 0.008, *p* = 0.095) (Table 1 and Fig. 3). These results were consistent with higher heme biosynthesis pathway expression associated with more severe OSA. The IV-exposure (heme biosynthesis pathway) and IV-outcome (OSA traits) associations in the discovery analysis are provided in Supplementary Table 2. The IVs were sufficiently strongly associated with the exposure in the IVW test ($\overline{F }$ = 52 > 10).

**Table 1:** Discovery two sample MR of canonical iron/heme related pathways (reactome) on OSA traits using Inverse Weighted Variance method.

Exposure	AHI				Average SpO_2_				Minimum SpO_2_				Sleep time% of SpO_2_ < 90%			
	nsnp	b	se	pval	nsnp	b	se	pval	nsnp	b	se	pval	nsnp	b	se	pval
Heme biosynthesis	29	0.007	0.005	0.189	30	− 0.008	0.005	0.095	30	− 0.008	0.005	0.078	30	0.012	0.004	6.14E − 03
Heme degradation	33	0.002	0.004	0.691	33	− 0.003	0.004	0.420	33	− 0.001	0.004	0.769	33	− 0.001	0.004	0.815
Heme signaling	128	− 0.001	0.003	0.819	129	− 0.002	0.003	0.580	129	− 0.003	0.003	0.301	129	0.001	0.003	0.761
Iron uptake and transport	138	− 0.004	0.003	0.191	138	0.001	0.003	0.729	138	0.002	0.002	0.335	138	− 0.001	0.002	0.534

We then performed MR Egger for this pathway to detect horizontal pleiotropy. The strength of instruments was sufficient for MR Egger regression (${I}_{GX}^{2}$ = 0.98 > 0.9). A moderate horizontal pleiotropy effect was found for AHI (MR Egger intercept *p* = 0.013; Table 2) but not for the other traits. The sensitivity analyses using MR Egger slope (i.e., considering the potential horizontal pleiotropy effects) for all four OSA traits showed consistent association directions with IVW analysis, suggesting that the putative association we observed was not driven by pleiotropic mechanisms (Table 2 and Fig. 3). The MR Egger slope test was significant for AHI, average SpO_2_, and sleep time% of SpO_2_ < 90% (*p* = 0.031, 0.030, and 0.018, respectively).

**Table 2:** MR sensitivity analysis between Reactome heme biosynthesis pathway and OSA traits.

Outcome	Method	Estimate	S.E.	*p* value	Heterogeneity statistic
AHI	IVW	0.007	0.005	0.189	Q = 27.979 (*p* = 0.412)
	MR egger intercept	0.030	0.011	0.013	
	MR egger slope	− 0.011	0.005	0.031	Q′ = 33.367 (*p* = 0.222)
	MR-PRESSO	0.007	0.005	0.200	
Average SpO_2_	IVW	− 0.008	0.005	0.095	Q = 25.307 (*p* = 0.611)
	MR egger intercept	0.008	0.005	0.095	
	MR egger slope	− 0.025	0.011	0.030	Q′ = 28.301 (*p* = 0.502)
	MR-PRESSO	− 0.008	0.005	0.102	
Minimum SpO_2_	IVW	− 0.008	0.005	0.078	Q = 31.140 (*p* = 0.311)
	MR egger intercept	0.004	0.005	0.384	
	MR egger slope	− 0.017	0.011	0.131	Q′ = 32.008 (*p* = 0.320)
	MR-PRESSO	− 0.008	0.005	0.089	
Sleep time% of SpO_2_ < 90%	IVW	0.012	0.004	0.006	Q = 30.966 (*p* = 0.319)
	MR egger intercept	− 0.006	0.004	0.157	
	MR egger slope	0.026	0.010	0.018	Q′ = 33.307 (*p* = 0.266)
	MR-PRESSO	0.012	0.004	0.010	

No significant heterogeneity of effects across the IVs were observed for any OSA trait (Q and Q′ *p* > 0.05 in Table 2). Leave-one-out IVW analysis showed consistent association for all traits (Supplementary Fig. 1). MR-PRESSO did not identify any IV outliers (Table 2). However, we observed heterogeneity effects across genes and tissues. Stronger effects of increased heme biosynthesis on increased OSA severity were observed in *FECH* and *UROD* genes, and in adiposity, esophagus mucosa, testis, lung, and artery tissues (see “Discussion”) (Supplementary Table 3, Supplementary Figs. 2 and 3).

Secondary analyses in GO pathways did not reveal any significant causal associations for OSA traits (*p* > 0.05; Supplementary Table 4).

### Validation MR

The MESA sample has 46.7% males, mean aged 68.6 years old and mean BMI 29.2 kg/m^2^, and 79.6% of the sample was classified with OSA (defined as AHI > 5) (Supplementary Table 5). The distributions of OSA traits and their correlations with demographic variables in MESA are shown in Supplementary Figs. 4 and 5. Similar to prior studies^20,21^, increasing AHI was observed with older age, male sex and higher BMI, and was highly correlated with all sleep-related hypoxemia measures (spearman correlation $\rho$ = − 0.57, − 0.73, and 0.76 with average, minimum SpO_2_, and sleep time% of SpO_2_ < 90%).

**Figure 4:**
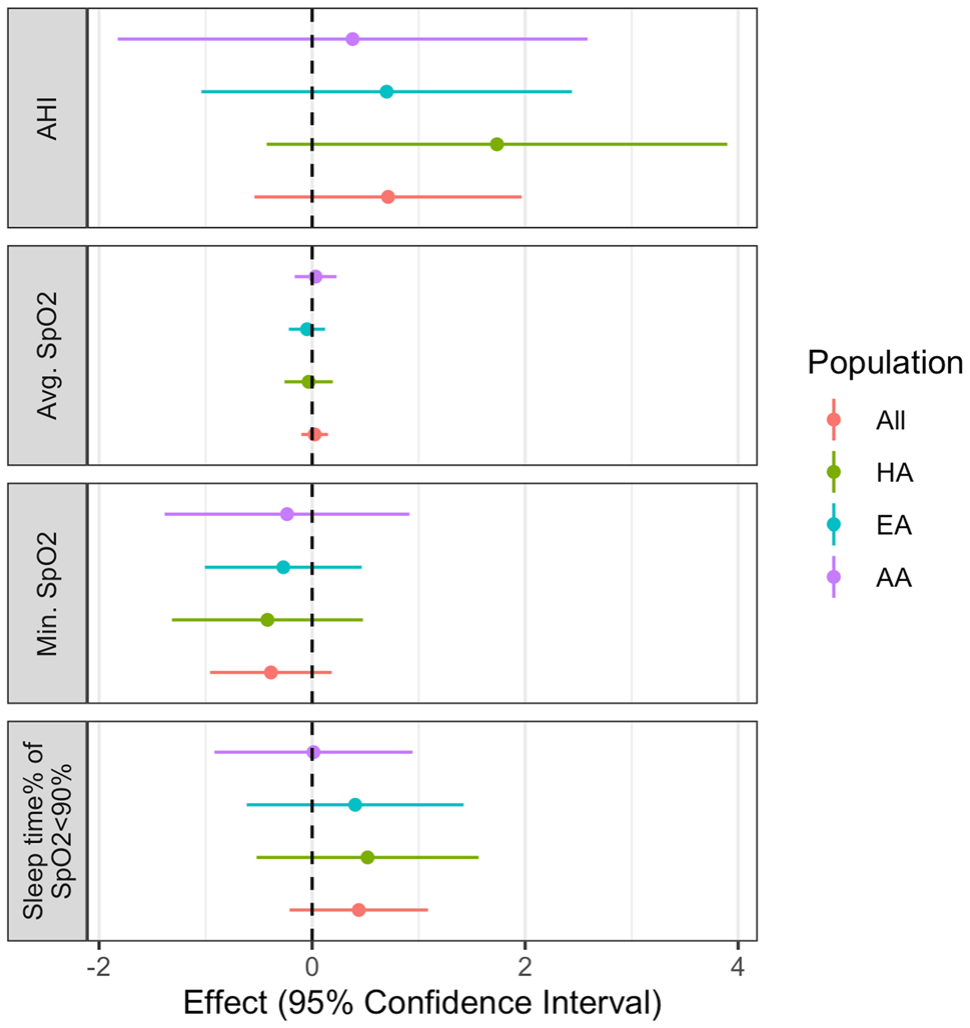
Validation MR analysis of the effect of upregulated Heme Biosynthetic Process pathway on OSA traits in MESA.

Although not statistically significant, we observed the same pattern for MR association directions between the increased heme biosynthesis expression with higher sleep time% of SpO_2_ < 90% ($\beta$ = 0.438, p = 0.187), lower minimum SpO_2_ ($\beta$ = − 0.387, p = 0.184), and higher AHI ($\beta$=0.713, p = 0.265) (Fig. 4 and Supplementary Table 6) as compared with the discovery analysis. In ancestry stratified analyses, we observed consistent directions (with the discovery MR analyses) for all OSA traits in EAs and HAs, but inconsistent directions of the associations for average SpO_2_ in AAs (Fig. 4 and Supplementary Table 6).

### MR association for heme biosynthesis pathway

To understand the biological function of the heme biosynthesis pathway, we performed two-sample MR between this pathway and other health outcomes. We identified a significant causal association between increased expression of the heme biosynthesis pathway with reduced risk of iron deficiency anemia (IVW $\beta$ = − 0.0003, *p* = 0.028 and MR-Egger $\beta$ = − 0.001, *p* = 0.027; Supplementary Table 6), consistent with prior knowledge^33^. We did not identify significant causal associations with specific blood cell traits or comorbid traits including obesity, asthma, type 2 diabetes, blood pressure, stroke, and cardiovascular, kidney, liver diseases (Supplementary Table 7).

## Discussion

This is the first MR analysis interrogating the causal association between molecular pathways and OSA. We implemented a novel pathway-based framework that leveraged information on gene expression and GWAS data, identifying a potential causal association between up-regulation in the heme biosynthesis pathway with increased OSA severity using publicly available data. This association is supported by sensitivity analyses and consistency of the point estimates in the validation analysis in a modestly sized sample with both gene expression and OSA measures.

Heme biosynthesis is a highly conserved process across species that forms heme (iron-protoporphyrin IX), a significant component of oxygen storage and transport proteins (e.g., hemoglobin, myoglobin) and enzymes involved in oxidative phosphorylation^34^, in the mitochondria and cytosol. Deficiency of heme synthesis enzymes results in the accumulation of intermediate porphyrins, which is associated with porphyria, a group of rare blood disorders characterized by various degrees of neurological deficits and photocutaneous lesions^35^. Lack of heme and hemoproteins are also associated with some types of anemia^35^. We were able to confirm a causal association between the increased expression of heme biosynthetic process pathway and decreased risk of iron deficiency anemia (Supplementary Table 6).

However, increased expression of heme synthesis enzymes may lead to excess heme. Cellular free heme is toxic and catalyzes the formation of reactive oxygen species, and thus increases oxidative stress^36^, which may augment the effects of intermittent hypoxia (a cardinal physiological manifestation of OSA), causing lung injury and subsequent gas exchange deficits, potentially leading to more severe sleep related hypoxemia in a setting of OSA^21^. In addition, heme may modulate ventilatory responses to hypoxia. In particular, heme degradation products (including carbon monoxide and biliverdin) have been shown to influence carotid body (the primary peripheral oxygen chemoreceptor) responses to hypoxia in rodent^7^, providing a ventilatory-related pathway linking heme to propensity for OSA. However, we did not detect any causal association between the heme degradation pathway with OSA traits. Future analyses using physiologically meaningful endotypes such as those that directly characterize hypoxia sensing^37–39^ and model organism research are needed to validate our hypothesis and rule out alternative mechanisms.

In this study, we observed some degree of heterogeneity of the MR associations across tissues (Supplementary Fig. 2). The largest effects of the upregulated heme biosynthesis pathway on increased OSA severity were observed using data from adipose tissue, testis, lung, esophagus mucosa, and artery tissues. Smaller effects were observed for data from whole blood. Validation analysis using expression data from blood cells therefore may be underpowered. Note that, the effect directions of heme biosynthesis on sleep time% of SpO_2_ < 90% (the most significant effect in our discovery analysis) is consistent across all tissues.

We also observed heterogeneity of the MR associations for heme biosynthesis on OSA across genes (Supplementary Fig. 3 and Supplementary Table 3). The largest effect of increased gene expression on increased OSA severity was observed for *FECH*, the last enzyme of this pathway that inserts iron to the protoporphyrin IX to generate heme. The effect direction is consistent with our prior admixture mapping results^40^. In the gene specific MR analyses for this pathway, we additionally identified potential causal associations for *uroporphyrinogen decarboxylase* (*UROD*). The increased expression of UROD was significantly associated with a more severe minimum SpO_2_ (IVW beta = − 0.042 and *p* = 0.004).

We also performed ancestry specific MR analyses in the MESA sample. We observed consistent directions of the associations in HA and EAs, but inconsistent directions for minimum SpO_2_ in AAs. This observation echoes our admixture mapping findings of *FECH* eQTLs which were driven by associations for higher AHI and sleep time% of SpO_2_ < 90% in European and Amerindian backgrounds^40^. While speculative, it is possible that there are distinct evolutionary pressures that influence the linkage between ventilatory and blood phenotypes across continents (e.g., reflecting adaptation to high altitude in Amerindians or to resistance to malaria in Africans)^41^.

This study has several strengths. The use of MR limits the unmeasured confounding bias and possible reverse causation between the instrument and the exposure because genetic variants are fixed at conception^11^. In the discovery analyses, we applied a well-powered two-sample MR analysis using publicly available summary statistics generated from a large gene expression database and OSA GWAS without need to access to original data. The two-sample design (no overlap between the exposure sample and outcome sample) further reduces the potential sample selection bias^22^. We implemented a set of sensitivity and heterogeneity analyses and validation analyses to interrogate the consistency of our findings in support of the potential causal association between heme biosynthesis pathway and OSA. The validity of our approach is supported by confirming a causal association between increased heme biosynthetic process expression and decreased risk of iron deficiency anemia (Supplementary Table 6).

This study also has several limitations. First, the effect sizes do not reflect the true effects of a potential clinical intervention. This is partly because MR estimates the cumulative effects of a lifelong exposure while the expression data were collected at a specific time point. It is also challenging to quantify pathway level expression across tissues in humans. Second, we were not able to formally confirm our findings in MESA (*p* > 0.05), which may be due to the insufficient statistical power in small samples, restriction to samples from peripheral blood cells, and heterogeneity effects across ancestry groups. However, we still observed the same association directions of point estimates in the validation analyses. Future analyses using experimental data from additional tissues (e.g., carotid body) may help elucidate more specific mechanisms. Analyses of a larger samples are needed to replicate our results. Third, given that we observed ancestry specific effects in the validation analyses, our primary analyses using multi-ancestry samples may reduce statistical power. Finally, due to the restricted use of cis-eQTL data (not GWAS of gene expressions), we did not test the reverse causality. Sustained hypoxia is associated with erythropoiesis and polycythemia^42^. Clinically evident differences in hematocrit levels are, however, rarely observed with OSA, but rather associate with sustained daytime hypoxemia rather than the intermittent nocturnal hypoxemia characteristics of OSA^43^. Nonetheless, a recent study showed that heme metabolism pathway is upregulated by intermittent hypoxia as shown by the results of continuous positive airway pressure (CPAP) withdrawal in OSA patients^10^. It is possible that there is bi-directional causal association between heme biosynthesis and OSA. While tracking erythropoietin levels has been suggested as a biomarker of OSA severity^44^, our data support the potential use of gene expression levels in heme biosynthesis pathways as a promising biomarker.

In conclusion, this study suggests a potential causal association between increased heme biosynthesis and OSA severity. Future work is needed to identify the mechanisms for this association, to address reverse causality, and evaluate utility of assessing gene expression of heme biosynthesis pathways as clinical biomarkers of disease susceptibility as well as disease severity.

## Supplementary Information

Supplementary Figures.

Supplementary Tables.

## Data Availability

The datasets used in the current study are available on dbGaP. Analysis code and intermediate results are available from the corresponding author on reasonable request.
