# Supplementary material for: Upregulated heme biosynthesis increases obstructive sleep apnea severity: a pathway-based Mendelian randomization study
Source: Sci Rep. 2022 Jan 27;12:1472. doi: 10.1038/s41598-022-05415-4 (PMC8795126; doi:10.1038/s41598-022-05415-4)

Upregulated heme biosynthesis increases obstructive sleep apnea severity: a pathway-based mendelian randomization study

Wang H et al.

Supplementary Fig. 1. MR leave-one-out analysis for the expression of heme biosynthesis pathway on OSA traits.

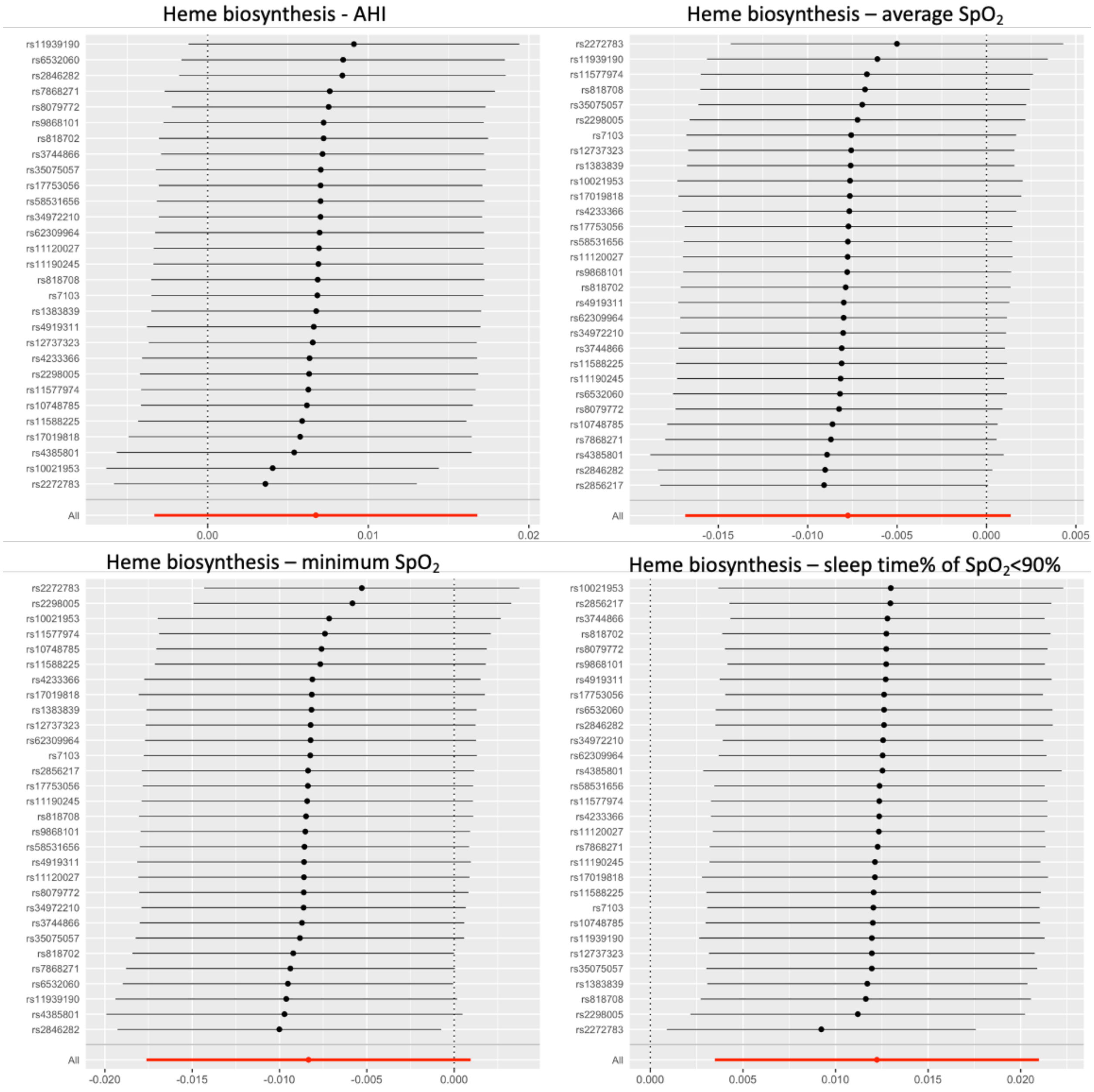

Supplementary Fig. 2. MR heterogeneity analysis across tissues for the expression of heme biosynthesis pathway on OSA traits.

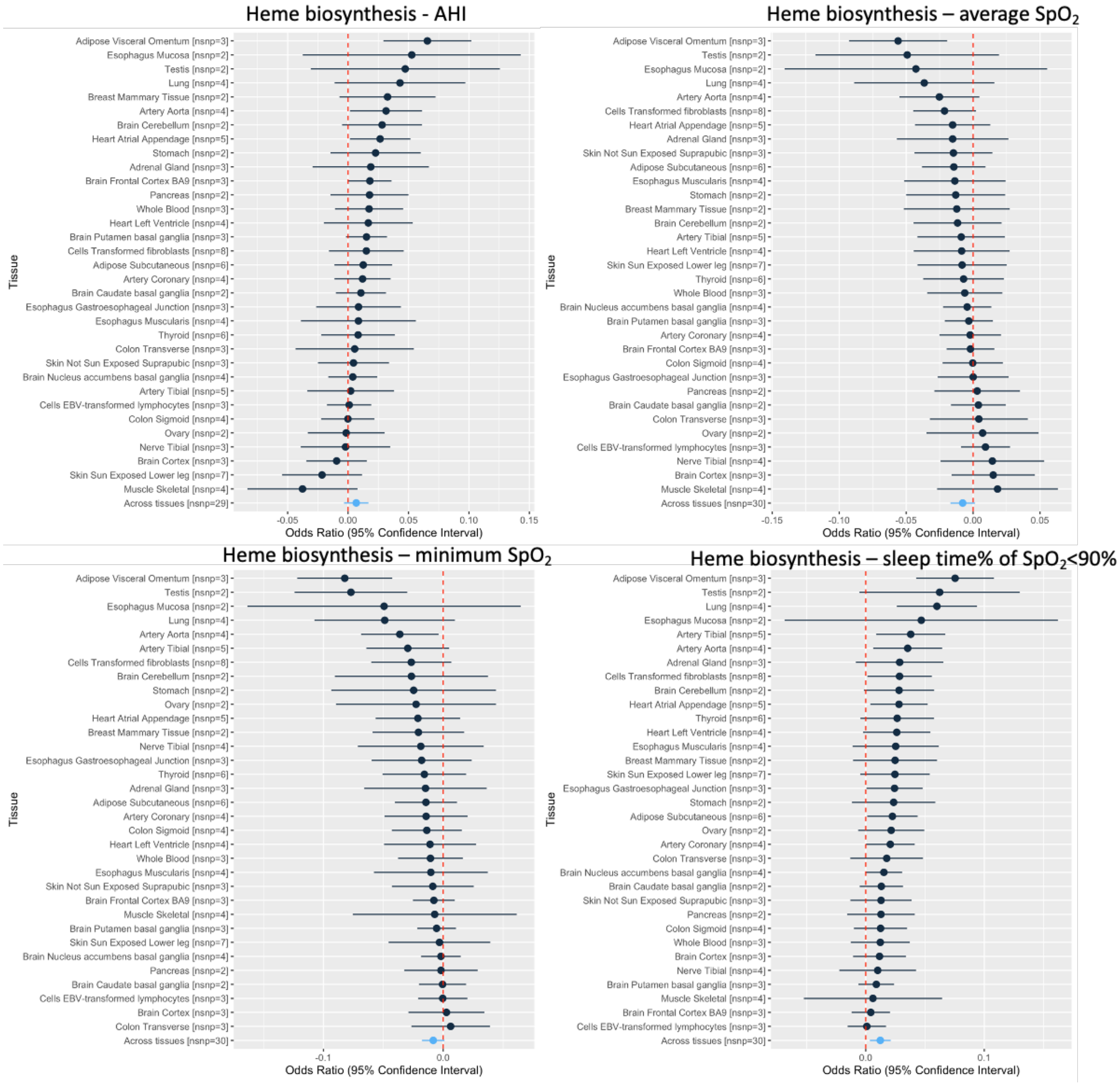

Supplementary Fig. 3. MR heterogeneity analysis across genes for the expression of heme biosynthesis pathway on OSA traits.

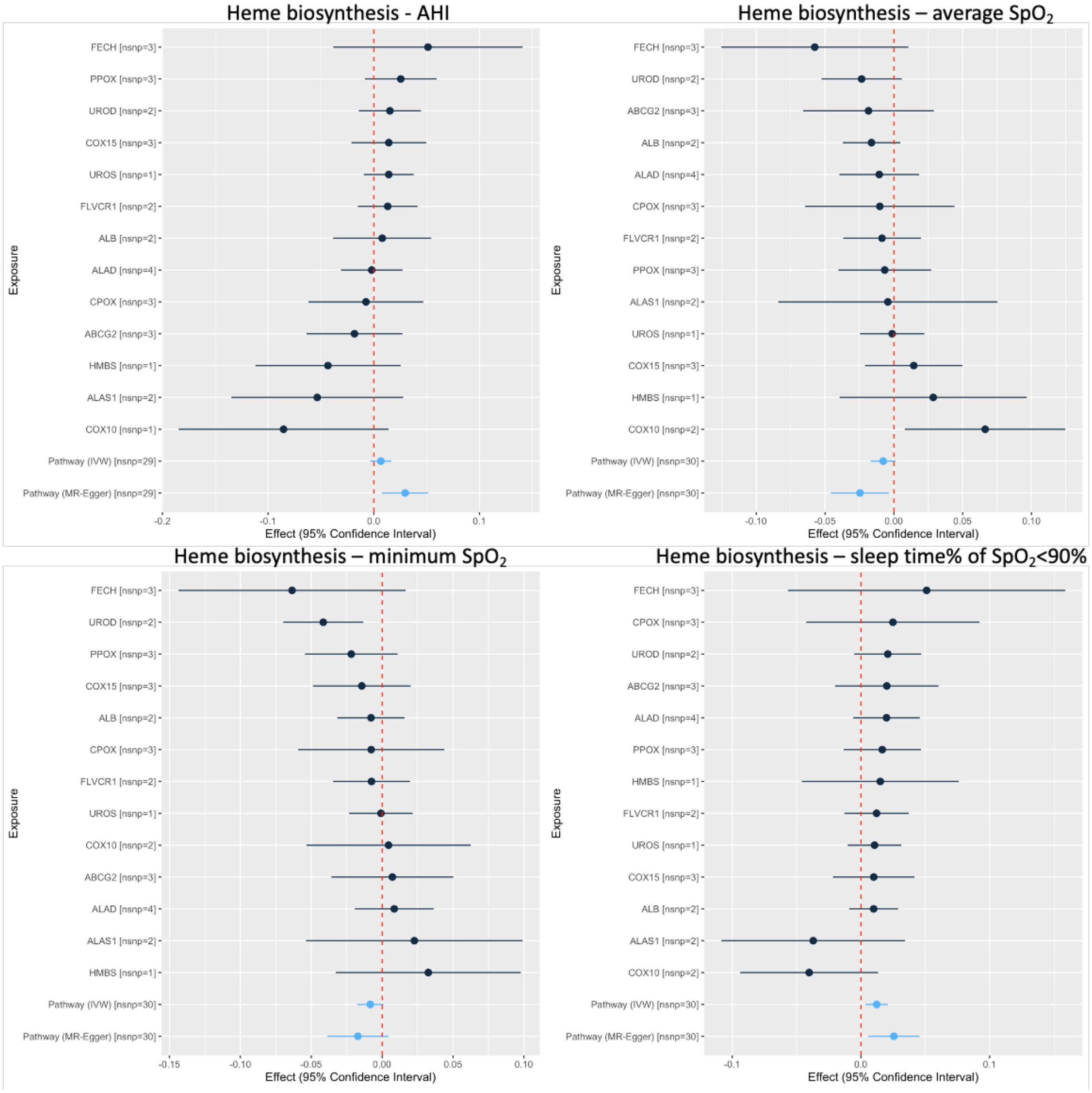

Supplementary Fig. 4. Distributions of OSA traits in MESA samples.

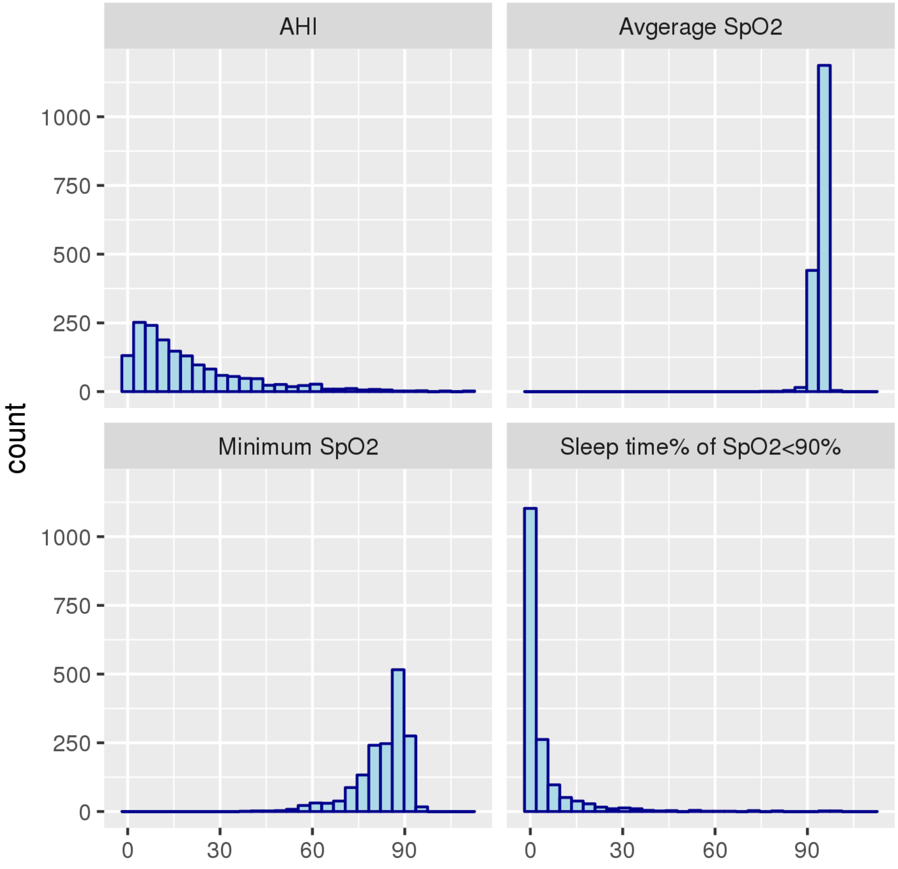

Supplementary Fig. 5. Spearman correlations between OSA traits and demographic variables in MESA samples.

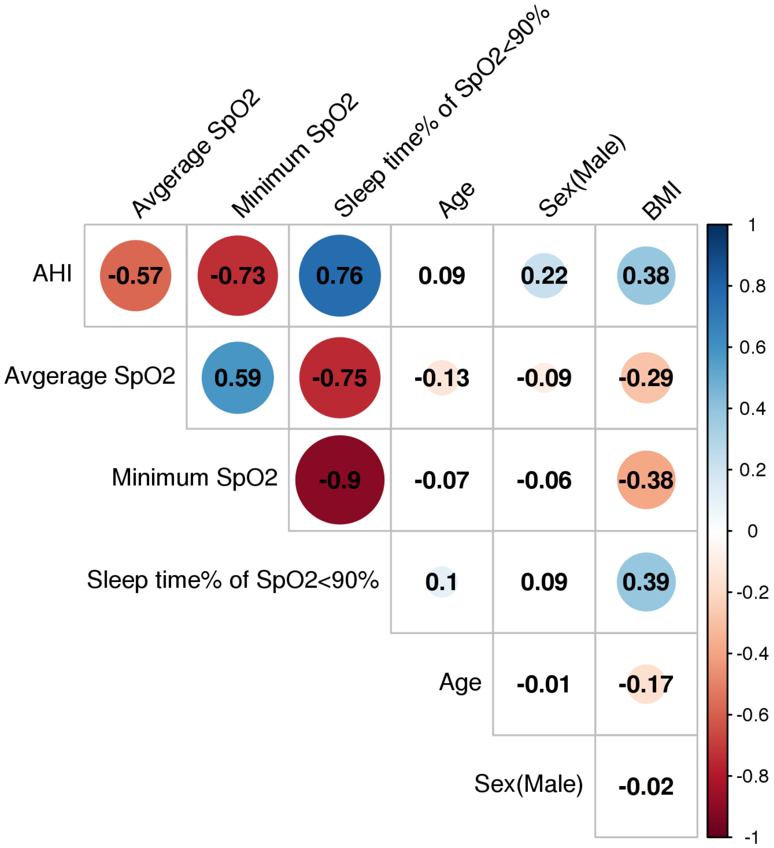

Supplement: Supplementary file 1 — Supplementary Figures. [file 41598_2022_5415_MOESM1_ESM.pdf]
